# Insight into the genome and brackish water adaptation strategies of toxic and bloom-forming Baltic Sea *Dolichospermum* sp. UHCC 0315

**DOI:** 10.1038/s41598-019-40883-1

**Published:** 2019-03-20

**Authors:** Jonna E. Teikari, Rafael V. Popin, Shengwei Hou, Matti Wahlsten, Wolfgang R. Hess, Kaarina Sivonen

**Affiliations:** 1 Department of Microbiology, University of Helsinki, Viikinkaari 9, FI-00014 Helsinki Finland; 2 Genetics & Experimental Bioinformatics, Institute of Biology III, University Freiburg, Schänzlestraße 1, D-79104 Freiburg, Germany

## Abstract

The Baltic Sea is a shallow basin of brackish water in which the spatial salinity gradient is one of the most important factors contributing to species distribution. The Baltic Sea is infamous for its annual cyanobacterial blooms comprised of *Nodularia spumigena*, *Aphanizomenon* spp., and *Dolichospermum* spp. that cause harm, especially for recreational users. To broaden our knowledge of the cyanobacterial adaptation strategies for brackish water environments, we sequenced the entire genome of *Dolichospermum* sp. UHCC 0315, a species occurring not only in freshwater environments but also in brackish water. Comparative genomics analyses revealed a close association with *Dolichospermum* sp. UHCC 0090 isolated from a lake in Finland. The genome closure of *Dolichospermum* sp. UHCC 0315 unraveled a mixture of two subtypes in the original culture, and subtypes exhibited distinct buoyancy phenotypes. Salinity less than 3 g L^−1^ NaCl enabled proper growth of *Dolichospermum* sp. UHCC 0315, whereas growth was arrested at moderate salinity (6 g L^−1^ NaCl). The concentrations of toxins, microcystins, increased at moderate salinity, whereas RNA sequencing data implied that *Dolichospermum* remodeled its primary metabolism in unfavorable high salinity. Based on our results, the predicted salinity decrease in the Baltic Sea may favor toxic blooms of *Dolichospermum* spp.

## Introduction

The Baltic Sea is one of the largest brackish water ecosystems in the world. It undergoes substantial spatiotemporal variation in the salinity gradient maintained by uneven saline-water inflow via the Danish Straits and varying amounts of riverine runoff. The surface-water salinity varies from >20 practical salinity units (PSUs) in Kattegat Bay to almost freshwater concentrations in the northernmost Gulf of Bothnia and eastern Gulf of Finland^1^. Climate change models predicted that the overall future salinity in the Baltic Sea will decrease, due to increased precipitation and freshwater inflow within the catchment area^2–4^. The salinity range is one of the most important factors contributing to the prevailing species diversity and distribution in this area^5–8^, and as a result of salinity decrease, the distribution of freshwater species may shift more to the south^2^. However, due to uneven saltwater pulses, organisms may occasionally become stressed by unfavorably high salt concentrations.

The Baltic Sea is notorious for its annual cyanobacterial blooms composed of *Nodularia spumigena*, *Aphanizomenon flos-aquae*, and *Dolichospermum* spp. (formerly *Anabaena* spp.)^9–11^. Blooms cause public-health concerns, especially for recreational users, because *Nodularia spumigena* and *Dolichospermum* spp. are capable of producing toxins^9,12^. The hepatotoxins nodularins and microcystins are detected in considerable amounts every summer and, despite general knowledge of their toxicity to mammals, animal poisonings are still reported regularly^13^.

*Dolichospermum* spp. is a filamentous and nitrogen-fixing cyanobacterial species that typically forms massive blooms in freshwater environments^14–16^. Furthermore, toxic strains capable of living in intermediate salinities have been found, especially in the less saline Gulf of Finland and coastal regions^11,17^. Decreased salinity may thus promote the distribution and abundance of *Dolichospermum* and further favor the formation of toxic *Dolichospermum* blooms^18,19^. Due to the genetic and morphological heterogeneity of the genus *Anabaena*, *Dolichospermum* was recently distinguished from a benthic *Anabaena*-type cluster, resulting in the gas vacuole-forming and pelagic morphotype *Dolichospermum* sp.^20^. However, *Dolichospermum* spp. still harbors high genetic and morphological diversity, which results in ambiguity in nomenclatural and phylogenetic clustering. Even mixing with *Aphanizomenon* spp. and *Nostoc* sp. has been described^21,22^. We have previously defined seven *Nostocales* subgroups (I a–d; II a–c), based on the comparison of average-nucleotide and amino-acid identities of 30 sequenced *Nostocales* strains^23^. In this classification, *Dolichospermum*/*Anabaena* species were found in four subgroups, implementing a high demand for further taxonomic clarification. However, a latter study suggested even more complex taxonomy due to the close relationship of these species to a third cyanobacterial genus, *Aphanizomenon*^24^.

Here, we sequenced the entire genome of Baltic Sea *Dolichospermum* sp. UHCC 0315 to reinforce the genomic knowledge of filamentous and toxic Baltic Sea cyanobacteria and to gain insight into the niche adaptation strategies of the examined strain. The isolate was obtained from a coastal cyanobacterial bloom in the Helsinki area^9^. Therefore, we addressed the question of how a slight increase in salinity relevant to the Gulf of Finland can affect the growth and construction of the transcriptomes. Furthermore, comparative genomic analysis was applied to elucidate the ambiguity within the sequenced *Dolichospermum*/*Anabaena* species.

## Results

### Insight into the genome of *Dolichospermum* sp. UHCC 0315

The whole genome sequence of *Dolichospermum* sp. UHCC 0315 was obtained using Pacific Biosciences (PacBio) chemistry, and sequencing errors were corrected with Illumina Hiseq2500 reads by two rounds of corrections (Freebayes 217 errors and Pilon 8 errors). The assembly yielded one chromosome of 5.17 Mbp and three plasmids (0.85, 0.53, and 0.94 Mbp). Automatic annotation of the *Dolichospermum* sp. UHCC 0315 genome revealed a total of 5,362 proteins of which 3,570 (66%) were predicted to be part of 367 metabolic subsystems. Among a wide diversity of functions, several response mechanisms to osmotic, oxidative and heat stress, as well as to antibiotic and toxic heavy metal defense were identified. Furthermore, one possible CRISPR array with 12 spacers and the direct repeat consensus sequence 5′-CTTGCAATTAACCTAATTACTCAAAGCTAATTTCACC-3′ but without any known Cas proteins was detected (Supplementary Fig. S1). Interestingly, spacer 7 matches prophage antirepressor sequences present in the genomes of other Nostocales, *Nostoc* ‘Peltigera membranacea cyanobiont’ N6 and *Nostoc flagelliforme* CCNUN1 (Supplementary Fig. S1), supporting the further function of this genetic element as a likely CRISPR repeat spacer array further.

**Figure 1:**
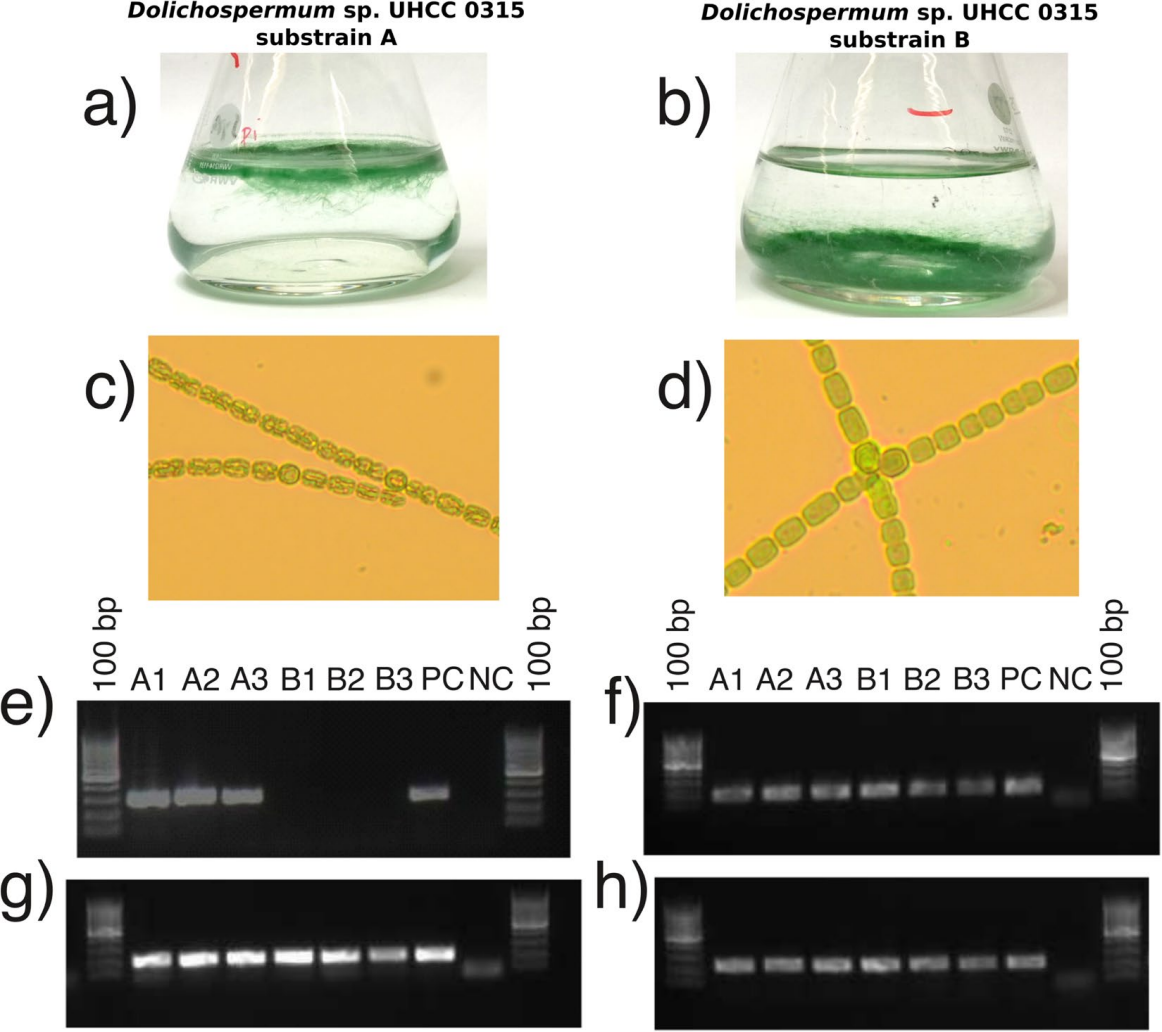
Identification of two *Dolichospermum* sp. UHCC 0315 substrains A and B. Subtype A showed buoyant phenotype (**a**) with rough morphology (presence of aerotopes) under the microscopy (**c**), whereas subtype B grew on the bottom of the cultivation flask (**b**) and had smooth morphology (**d**). The PCR amplification study using specific primers and testing triplicates (**e**) further showed that the genetic region of BMF77_1250–1254 is present in substrain A (A1–3) but absent in B (B1–3). However, the results indicate that both substrains harbor full sets of three plasmids (**f–h**). PC = Positive Control; NC = Negative Control. The full-length gel with the PCR amplification of the three plasmids are presented in Supplementary Figs S2 (**e**) and S3 (**f–h**).

Interestingly, the assembly of the *Dolichospermum* sp. UHCC 0315 genome unraveled two chromosomal subtypes, A and B, of which subtype A carried an additional five adjacent hypothetical genes (BMF77_1250–1254). Based on the Protein Basic Local Alignment Search Tool (BlastP) search, these five genes encode a putative methyltransferase (BMF77_01250), glycosyltransferase (BMF77_01251; BMF77_01253; BMF77_01254), and a hypothetical protein (BMF77_01252). PacBio coverages of the two identified subtypes were 250 (subtype A) and 70 (subtype B). To ensure that both subtypes were properly distinguished by sequence assembly, we isolated 70 filaments under a microscope and cultivated them in liquid medium. Surprisingly 39 of the cultures were buoyant (subtype A) and 31 grew on the bottom of the flask (subtype B) (Fig. 1a,b), despite both subtypes carried full *gvp* gene cluster for gas vesicle formation. Differences in cellular morphology under the light microscope were additionally observed and indicate the presence of aerotopes in substrain A (Fig. 1c,d). Presence or absence of the 5-gene region was  confirmed by PCR amplification of a segment from gene BMF77_1254 using specific primers (Supplementary Table S1). The amplification product was found only in the buoyant subtype A (Fig. 1e, Supplementary Fig. S2), which further showed that the absence of the BMF77_01250–01254 genes in subtype B was real. Furthermore, the distribution of all three plasmids was tested, using primers amplifying certain regions of the plasmids, and the PCR results indicated that all three plasmids were present in both substrains (Fig. 1f–h, Supplementary Fig. S3).

### Comparative genomics

**Figure 2:**
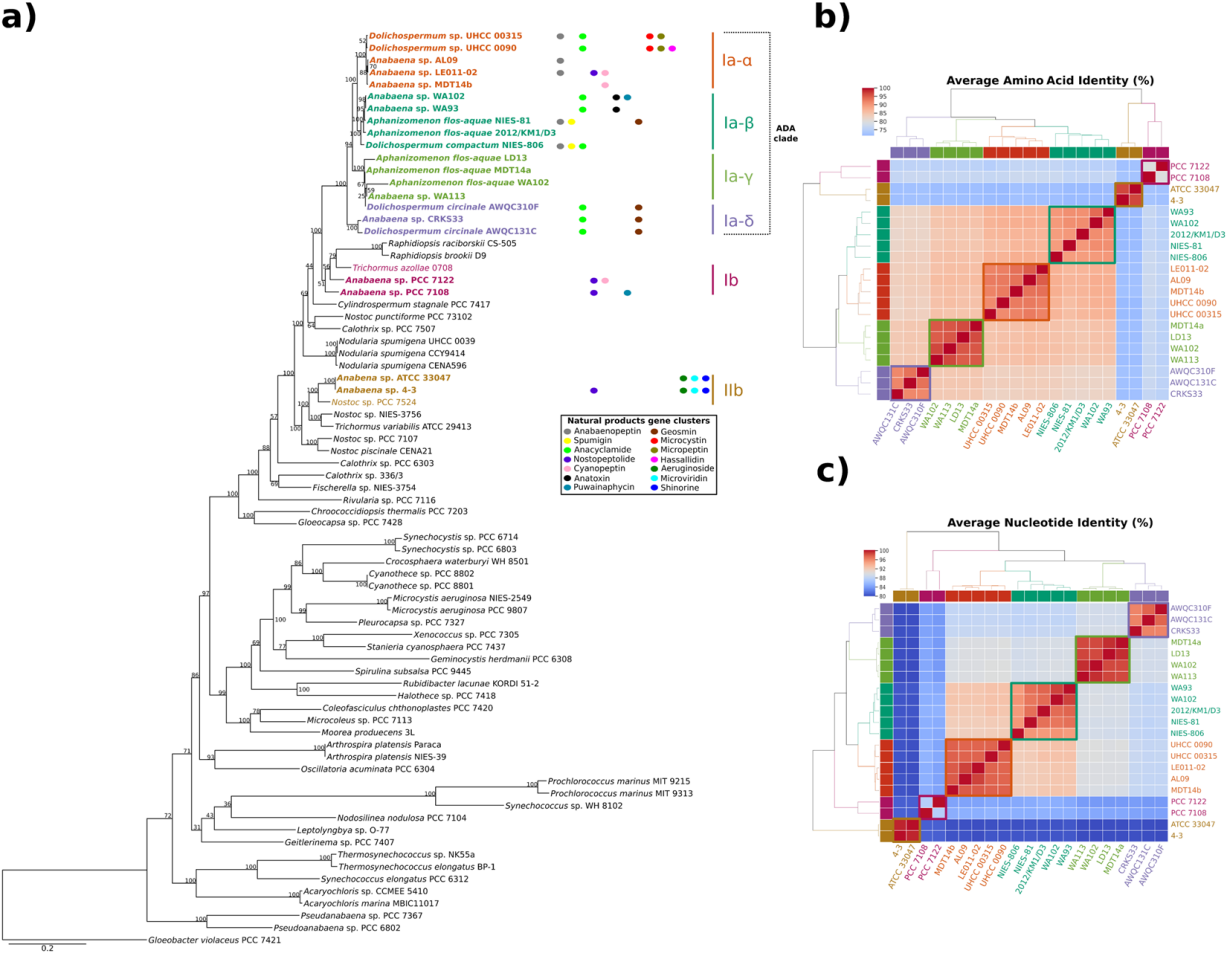
Maximum likelihood phylogenomic tree based on the concatenated alignment of 31 universal marker genes^70^ from 75 cyanobacterial genomes. Those analyzed in this study are shown in bold and the presence of gene clusters of natural products are represented (**a**). Average Amino Acid Identity (AAI) (**b**), and Average Nucleotide Identity (ANI) (**c**) heatmaps of genomes in subgroups (Iα-δ, II and III). Subgroups were determined as described earlier^23,24^.

A phylogenomic tree, based on 31 conserved marker genes was constructed to show the placement of the newly sequenced *Dolichospermum* sp. UHCC 0315 within the cyanobacterial phylum (Fig. 2a). The strain branched together with 16 other *Dolichospermum* sp., *Anabaena* sp. and *Aphanizomenon flos-aquae* with diverse biosynthetic gene clusters, forming a distinct *Anabaena/Dolichospermum/Aphanizomenon* clade (ADA)^24^. Other 4 *Anabaena* strains (PCC 7122, PCC 7108. ATCC 33047 and 4-3) formed different branches in the tree. Furthermore, Average Amino Acid and Nucleotide Identity analyses (Fig. 2b,c, respectively) highlighted the division of the genomes in 6 different subclades (Iα-δ, II and III). Considering the genome of *Dolichospermum* sp. UHCC 0315, there are five genomes of the genus that are publicly available (*Dolichospermum circinale* AWQC310F and AWQC131C and *Dolichospermum* sp. NIES-806 and UHCC 0090). However, while only the UHCC 0315 and UHCC 0090 strains grouped together in a subclade with other 3 *Anabaena* strains (AL09, LE011-02 and MDT14b) in the genomic analyses, the other ones were scattered in other subclades. A 16S rRNA phylogenetic tree was constructed to further explore the ADA clade (Supplementary Fig. S4).

**Figure 3:**
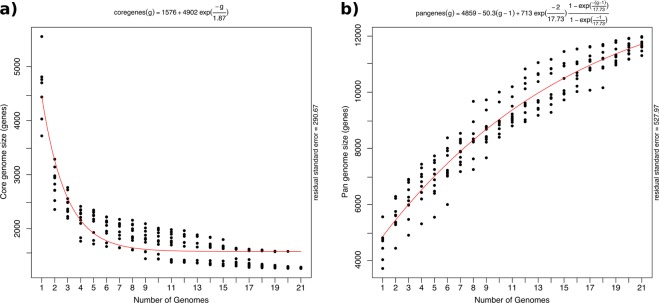
Core and pan-genome plots estimated using the ADA clade (*Anabaena* sp. WA102, WA93, AL09, LE011-02, MDT14b, WA113 and CRKS33; *Aphanizomenon flos-aquae* NIES-81, 2012/KM1/D3, LD13, MDT14a and WA102; *Dolichospermum* sp. NIES-86, UHCC00315, UHCC 0090; and *Dolichospermum circinale* AWQC310F and AWQC131C plus *Anabaena* sp. PCC 7122, PCC7108, ATCC 33047 and 4-3. (**a**) Number of genes in the core-genome size by the number of genomes and (**b**) number of genes in the pan-genome by the number of genomes.

Orthologous gene clustering was employed to estimate the core and pan-genome of the 21 *Anabaena*, *Dolichospermum* and *Aphanizomenon* genomes (Fig. 3). Whereas the core-genome asymptote is composed of approximately 1,500 genes, the current pan-genome reached more than 12,000 different genes belonging to the ADA clade.

The closest relative to *Dolichospermum* sp. UHCC 00315 was the freshwater strain *Dolichospermum* sp. UHCC 0090 (previously *Anabaena* sp. 90). Synteny analysis showed a high number of conserved blocks between the two strains in the chromosomes and the pDOL01 and pUHCC0315a plasmids. Nonetheless, the other two plasmids of each strain seemed to be considerably less conserved (Supplementary Fig. S5). The two genomes encode a total of 9,873 proteins of which 7,101 formed shared orthologous clusters (Supplementary Fig. S6). Around 93% of the proteins in those clusters were predicted to be involved in a wide diversity of functions of the cellular primary and secondary metabolisms. On the other hand, 1,816 and 956 proteins were found to be specific to *Dolichospermum* sp. UHCC 0315 and UHCC 0090, respectively (21.19% and 33,86% of the total number of proteins encoded in each genome). Functions for these two groups of proteins were less successfully predicted compared to the shared genes, pointing to many previously unknown genes among them.

### Growth of *Dolichospermum* sp. UHCC 0315

**Figure 4:**
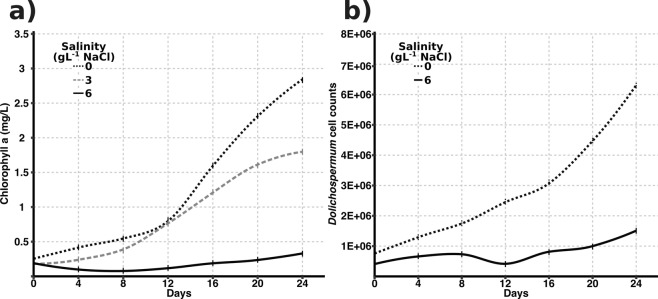
Growth of *Dolichospermum* sp. UHCC 0315 at different salinities. Growth was followed during the experiment of 24 days by chlorophyll *a* determination (**a**) and cell counting (**b**).

*Dolichospermum* sp. UHCC 0315 growth rate was followed at four different salinities (0, 3, 6, or 9 g L^−1^ NaCl) by measuring the chlorophyll *a* concentration (Fig. 4a). The highest tested salinity (9 g L^−1^ NaCl) was deleterious for *Dolichospermum* sp. UHCC 0315, while 6 g L^−1^ NaCl inhibited the growth of the culture. *Dolichospermum* sp. UHCC 0315 was able to grow properly in 3 g L^−1^ NaCl, but the fastest growth was obtained under freshwater conditions (no added NaCl). Due to the appearance of a yellowish color in the culture, cell counting using the microscope technique was applied to follow the growth (Fig. 4b). The patterns of chlorophyll *a* and cell counting seemed to correlate, and thus the concentration of chlorophyll *a* was later used for normalization of the toxin concentration.

### Reconstruction of transcriptomes under unfavorable salinities

**Figure 5:**
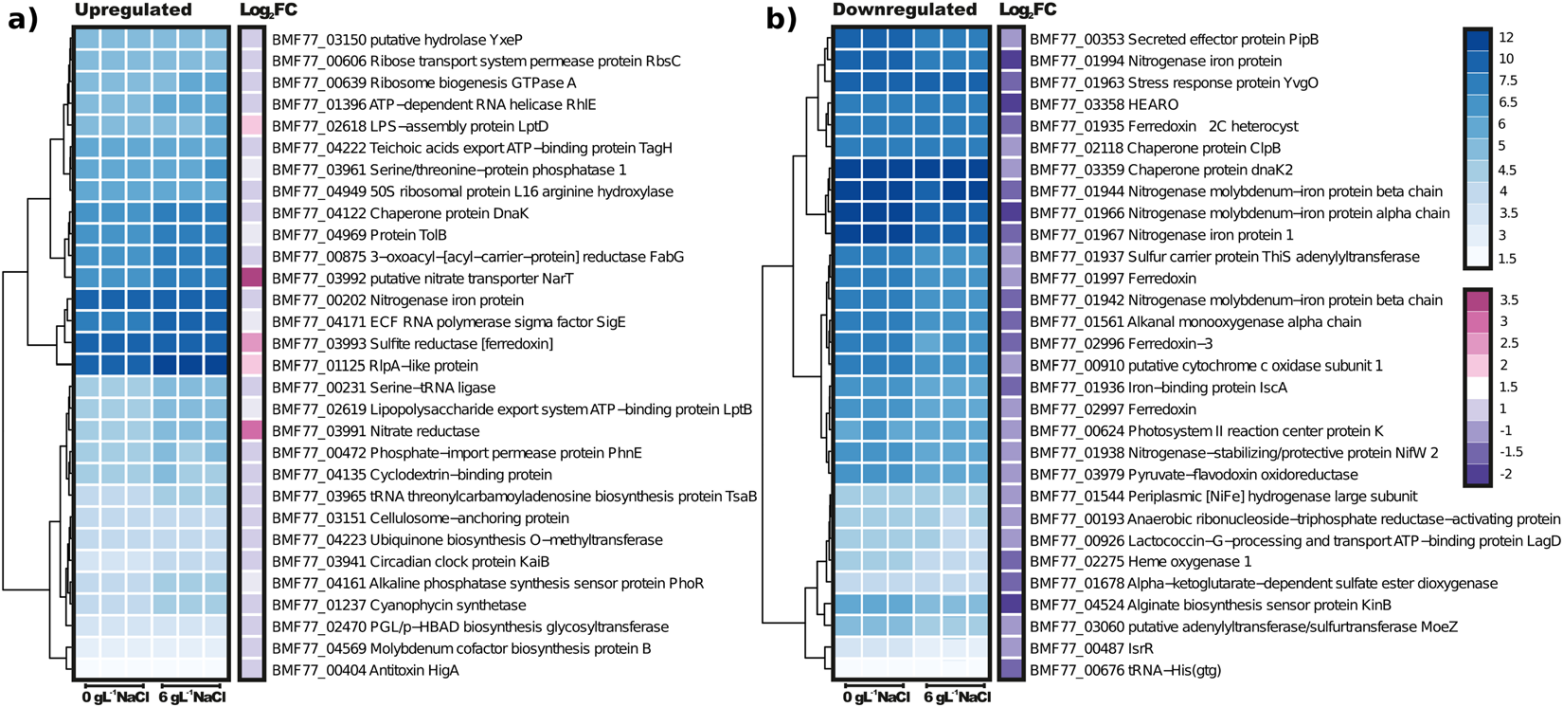
Heatmaps presenting Log2 Fold Changes of 30 most upregulated (**a**) and downregulated (**b**) genes in triplicates at two different salinities (0 g L^−1^, and 6 g L^−1^, of added NaCl).

To understand the cellular response of *Dolichospermum* sp. UHCC 0315 at moderate salinity, we applied RNA sequencing (RNA-Seq) analysis to compare the normal (0 g L^−1^ NaCl) and unfavorable conditions (6 g L^−1^ NaCl). We mapped a total of 4.1 × 10^8^ sequenced paired-end Illumina HiSeq reads for three biological replicates of the control and 4.2 × 10^8^ for three biological replicates of the treatment against the sequenced reference genome. Since the *Dolichospermum* sp. UHCC 0315 B lacked five genes and the RNA-seq data was generated from the mixture of the subcultures, *Dolichospermum* sp. UHCC 0315 A was used as a reference genome for mapping and calling of the differentially expressed genes. Using a Log_2_ fold change (Log_2_FC) of ±1 and a False-Discovery Rate (FDR) cutoff value < 0.01 provided by DESeq software^25^, the number of differentially expressed genes for *Dolichospermum* sp. UHCC 0315 was 348, of which 197 genes were upregulated and 151 were downregulated (Supplementary Table S2). A total of 136 upregulated and 90 downregulated genes were classified as hypothetical proteins or proteins with unknown functions, showing an extensive gap in our knowledge of the metabolism and genetic reservoir of the examined cyanobacteria. *Dolichospermum* sp. UHCC 0315 was struggling in the unfavorable environment of moderate salinity, suppressing many metabolic processes and protecting cellular biomolecules (Supplementary Fig. S7). The high demand for metabolism remodeling was implemented by adjusting the gene expression of transcription and translation, including 10 upregulated and two downregulated genes (Table 1). Furthermore, the pool of chaperones was reorganized. Nitrogen fixation is energetically expensive for cyanobacteria and pathway seemed to be mainly repressed (BMF77_01942-3; BMF77_01966-67: BMF77_01994-96) despite one nitrogenase gene was upregulated (BMF77_00202). Moreover, the nitrogen requirement was most likely fulfilled by reorganizing existing nitrogen reservoirs e.g. as indicated by the induced expression of BMF77_01290, nitrate reduction (BMF77_3991; 3993), and fine-tuning amino-acid metabolism (BMF77_00644; 02305; 03528). High demand for the reorganization of nitrogen metabolism was also seen among 30 most up and downregulated genes (Fig. 5). A general stress response was implemented, hindering the expression of the genes associated with photosynthesis (BMF77_00624; 00701; 01361; 02844; 02798) and electron transfer (BMF77_00909; 01935; 01997; 02996-7; 03623). In addition, induction of genes involved in modifying cell-wall components or encoding multiple transporters illustrated a need for reorganization of the cell-wall structure. In freshwater cyanobacteria, trehalose and sucrose are the major compatible solutes. Sucrose synthase genes were found in *Dolichospermum* UHCC 0315 but, surprisingly, there was no differential gene expression detected for the sucrose synthase genes. Hence, the absence of certain genes for compatible solute synthesis and the lacking induction of certain genes are probably important factors limiting the acclimation of *Dolichospermum* UHCC 0315 to higher salinity.

**Table 1:** List of the selected differentially expressed genes of *Dolichospermum* sp.

Gene	Product	Log2 FC
**Photosynthesis and electron transport**		
BMF77_02844	Allophycocyanin alpha chain	−1,17
BMF77_02798	Photosystem I reaction center subunit PsaK	−1,10
BMF77_01361	Photosystem II protein Y	−1,03
BMF77_00624	Photosystem II reaction center protein K	−1,28
BMF77_00701	Phycobilisome rod-core linker polypeptide CpcG4	−1,13
BMF77_00909	Cytochrome c oxidase subunit 2	−1,05
BMF77_02997	Ferredoxin	−1,89
BMF77_01997	Ferredoxin	−1,38
BMF77_01935	Ferredoxin, heterocyst	−1,57
BMF77_02996	Ferredoxin-3	−1,79
BMF77_03623	Plastocyanin	−1,25
**Nitrogen cycle**		
BMF77_04749	2-isopropylmalate synthase	−1,21
BMF77_01994	Nitrogenase iron protein	−2,00
BMF77_01967	Nitrogenase iron protein 1	−1,98
BMF77_01966	Nitrogenase molybdenum-iron protein alpha chain	−2,21
BMF77_01944	Nitrogenase molybdenum-iron protein beta chain	−1,95
BMF77_01942	Nitrogenase molybdenum-iron protein beta chain	−1,50
BMF77_01938	Nitrogenase-stabilizing/protective protein NifW 2	−1,41
BMF77_03247	Global nitrogen regulator	−1,04
BMF77_03993	Sulfite reductase [ferredoxin]	2,86
BMF77_03991	Nitrate reductase	3,37
BMF77_00202	Nitrogenase iron protein	1,32
**Amino acid metabolism**		
BMF77_03528	Glutamate racemase	1,12
BMF77_02305	LL-diaminopimelate aminotransferase	1,06
BMF77_00605	High-affinity branched-chain amino acid transport system permease protein LivH	1,10
BMF77_01290	Putative serine protease HtrA	1,01
BMF77_00644	Aspartate aminotransferase	−1,20
BMF77_01666	Cystathionine beta-lyase MetC	−1,04
BMF77_01996	Cysteine desulfurase NifS	−1,22
BMF77_01034	ATP-dependent Clp protease ATP-binding subunit ClpC	−1,08
**Transcription and translation**		
BMF77_04949	50 S ribosomal protein L16 arginine hydroxylase	1,18
BMF77_01396	ATP-dependent RNA helicase RhlE	1,42
BMF77_04171	ECF RNA polymerase sigma factor SigE	1,52
BMF77_04319	NADPH-dependent 7-cyano-7-deazaguanine reductase	1,11
BMF77_02346	putative dual-specificity RNA methyltransferase RlmN	1,10
BMF77_00639	Ribosome biogenesis GTPase A	1,21
BMF77_04465	RNA polymerase sigma factor SigA	1,01
BMF77_00231	Serine–tRNA ligase	1,36
BMF77_02360	SsrA-binding protein	1,04
BMF77_03965	tRNA threonylcarbamoyladenosine biosynthesis protein TsaB	1,38
BMF77_02233	tRNA pseudouridine synthase A	−1,02
BMF77_00676	tRNA-His(gtg)	−1,79
**Chaperones**		
BMF77_03414	10 kDa chaperonin	−1,17
BMF77_02118	Chaperone protein ClpB	−1,35
BMF77_03359	Chaperone protein DnaK2	−1,39
BMF77_00729	Chaperone protein DnaK	1,01
BMF77_04122	Chaperone protein DnaK	1,19
**Cell wall and lipids**		
BMF77_02633	Malonyl CoA-acyl carrier protein transacylase	1,14
BMF77_03151	Cellulosome-anchoring protein	1,44
BMF77_02469	TVP38/TMEM64 family inner membrane protein YdjZ	1,09
BMF77_02020	Murein DD-endopeptidase MepS/Murein LD-carboxypeptidase	1,10

UHCC 0315 in moderate salinity (6 g L-1 NaCl). Negative Log2 Fold Change values showed downregulated genes whereas positive values show up-regulated genes.

### Biosynthetic potential

**Figure 6:**
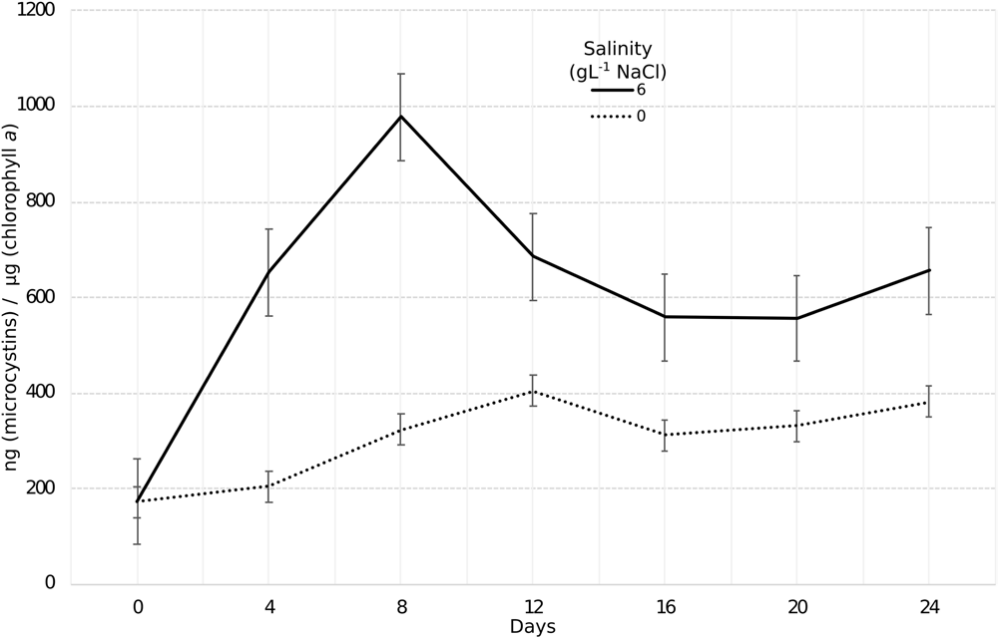
Amount of microcystins normalized against chlorophyll *a* concentration produced by *Dolichospermum* sp. UHCC 0315 during the experiment of 24 days. The salinities refer to the added NaCl.

Based on antiSMASH analysis, the genome of *Dolichospermum* sp. UHCC 0315 harbors six known and four unknown gene clusters for the synthesis of naturally bioactive metabolites (Fig. 1a, Supplementary Table S3). Due to the capability of the strain to produce microcystins, our next interest was to follow the toxin concentration at freshwater and moderate salinities. At the latter salinity, the microcystin gene cluster was slightly upregulated (Table 2). Interestingly, the normalized microcystin concentration increased at unfavorably high salinities, reaching the highest amount at day 8 (892.4 ng microcystin/µg chlorophyll, Fig. 6). After that time, the concentration decreased slightly and approached the toxin level of the control cultures during the later phase of the experiment, whereas total quota of the microcystin concentration in the system increased during the experiment (Supplementary Fig. S8a). Combined intra- and extracellular microcystin concentrations were measured every fourth day at 0 and 6 g L^−1^ NaCl (Supplementary Fig. S8b).

**Table 2:** Expression of the genes in the microcystin synthetase (*mcy*) gene cluster.

Gene	Gene name	Product	Log2 FC	FDR
BMF77_03385	*mcyC*	non-ribosomal peptide synthetase	0,53	7,36E-09
BMF77_03386	*mcyB*	non-ribosomal peptide synthetase	0,26	2,54E-02
BMF77_03387	*mcyA*	McyA protein	0,27	1,81E-02
BMF77_03388	*mcyG*	peptide synthetase polyketide synthase fusion protein McyG	0,36	1,14E-03
BMF77_03389	*mcyD*	polyketide synthase	0,38	4,77E-05
BMF77_03390	*mcyJ*	methyltransferase	0,30	2,33E-03
BMF77_03391	*mcyE*	hybrid non-ribosomal peptide synthase/polyketide synthase	0,41	8,97E-05
BMF77_03392	*mcyF*	Asp/Glu racemase McyF	0,56	1,43E-06
BMF77_03393	*mcyI*	dehydrogenase McyI	0,44	3,37E-07
BMF77_03394	*mcyH*	ABC transporter ATP-binding protein	0,31	2,00E-02

Positive Log2Fold Change values showed that the whole *mcy* gene cluster was slightly significantly upregulated in moderate salinity (6 g L^−1^ NaCl). Log2 FC = Log2 fold change. FDR = False discovery rate.

## Discussion

Species diversity in brackish water ecosystems is usually relatively narrow, because most of the organisms are adapted to live in either a marine or freshwater environment^7^. A recent study showed that *Nodularia spumigena* has remarkable genomic plasticity in acclimation strategies to the brackish water Baltic Sea^23^. However, in brackish water regions, *N*. *spumigena* coexisted with toxic *Dolichospermum* spp. but the former managed to sense the higher salinities fundamentally better than in freshwater environments^23^. In the light of projected salinity decreases in the Baltic Sea area, the question of whether freshwater origin *Dolichospermum* spp. may predominate in future cyanobacterial blooms in the Baltic Sea has arisen. Cyanobacteria from this genus are filamentous, capable of fixing atmospheric nitrogen, usually produce toxins, conditionally buoyant, and form akinetes under unfavorable conditions^24,26^. The abundance of *Dolichospermum* in the Baltic Sea is thus a consequence of ecological niche adaptation, for which the genomic inventory and regulatory apparatus provide the genetic underpinning.

Two substrains of *Dolichospermum* sp. UHCC 0315 (A and B) were distinguished at the genomic and physiological levels. Substrain B lacked a region of five genes, whereas substrain A harbored these genes and was capable of buoyancy. Otherwise, the genomic structure, including the same combination of plasmids, was similar albeit information on single-nucleotide polymorphisms (SNPs) was not gained by the methods used in this study. The association between the genes and the observed buoyancy phenotype is not directly clear, because both subtypes carry the full set of genes for gas vesicle formation (*gvpA*, *gvpC*, *gvpN*, *gvpJ*, *gvpK*, *gvpF*, *gvpG*, *gvpV*, *gvpW*. BMF77_4471–4480), similar to *Nostoc* sp. PCC7120 and *Dolichospermum* sp. UHCC 0090^27,28^. Moreover, SNPs may have a great impact on the gene function. Even though the buoyancy of the cyanobacteria can be controlled by mechanisms other than gas vesicles^29^, the functions tentatively assigned to these five genes point towards the modification of surface properties, so a link, even if circumstantial, with buoyancy cannot be excluded. It is unknown when these changes occurred, during the years in the culture collection or before. However, stable laboratory conditions force cyanobacteria to diminish their energy demand by eliminating functionally redundant and nonessential parts of the genome that enhance fitness in the environment but are unnecessary under stable laboratory conditions^28^. Moreover, water column vertical migration and co-occurrence of buoyant/non-buoyant populations of cyanobacteria have been documented as result of variations in light incidence and nutrient concentration^30^. Identification of two substrains in the same culture underpins the importance of whole-genome analysis to elucidate explicit genetic structure and enhance the knowledge of novel genetic elements. The method coupling the short-read Illumina and long-read PacBio sequences used in this study serves as a platform for sequencing plastic genomes with high numbers of repetitive elements.

The genus *Anabaena* was recently separated into two genera: benthic *Anabaena* and gas-vesicle-producing planktonic *Dolichospermum*^20^. However, the new nomenclature has not yet been fully adopted by the scientific community, and mixing between these two genera and even *Aphanizomenon* and *Nostoc* species still occurs^9,17^. Nonetheless, a recent study indicates that *Dolichospermum/Anabaena* possess closely related genomic features to *Aphanizomenon flos-aquae*^24^. Considering that only *Aphanizomenon flos-aquae* isolates have been analyzed, future studies with other species, such as *A*. *gracile and A*. *issatschenkoi*, could uncover whether the entire genus is related to *Anabaena* and *Dolichospermum*. In the phylogenomic tree, the newly sequenced *Dolichospermum* sp. UHCC 0315 branched with several other members of the *Anabaena/Dolichospermum/Aphanizomenon* clade (ADA), while *N*. *spumigena* was separated into a single tight cluster in accordance to previous results^23^. However, 4 *Anabaena* strains (PCC 7122, PCC 7108. ATCC 33047 and 4-3) genomes formed 2 divergent clades as previously shown^23^ and might indicate a yet not fully understood taxonomic relationship. In the case of strains ATCC 33047 and 4-3, the taxonomy seems to be even more unclear since 16S rRNA allocated these two strains among *Nostoc*, *Desmonostoc* and *Aliinostoc*. The inclusion of new genomes of these cyanobacteria and further taxonomic studies will possibly indicate whether the ADA clade is polyphyletic or should these four *Anabaena* genomes be reclassified in a different taxon. As expected, the addition of *Dolichospermum* sp. UHCC 0315 to the ADA group core-genome analysis did not affect the previously estimated common gene pool of 1,500 genes^24^. However, the pan-genome of the clade is yet open and calculated to be currently similar to the one for *Microcystis* (12,000)^31^. Therefore, future inclusions of new genomes are likely to expand the full complement of genes of the clade.

The genome size of newly sequenced *Dolichospermum* sp. UHCC 0315 was 5.4 Mbp, which is approximately the same size as its closest counterpart *Dolichospermum* sp. UHCC 0090 (5.31 Mbp)^28^. Moreover, the comparison of the two complete genomes indicated a relatively high level of conservation between them and no clear divergence of biosynthetic potential or coding sequences. Therefore, the close genetic association of UHCC 0315 with a freshwater cyanobacteria and the harmfulness of small increases in salinity indicate that *Dolichospermum* spp. have most probably been transported from freshwater ecosystems to the Baltic Sea.

The most rapid growth of *Dolichospermum* sp. UHCC 0315 was obtained under freshwater conditions (0 g L^−1^ NaCl), while higher salinities (>3 g L^−1^ NaCl) clearly hindered growth. However, slight increases in salinity (3 g L^−1^ NaCl) were not harmful for the strain, suggesting that the examined cyanobacterium sensed minor increases in salinity in studied environments, which may be caused by water fluctuation and saltwater pulses. Our findings agree well with field studies in the Baltic Sea in which salt concentrations play crucial roles in the abundance and intensity of *N*. *spumigena* and *Dolichospermum* spp. in cyanobacterial blooms^19,23^. Furthermore, the abundance of *Dolichospermum* is higher in the low-salinity coastal regions, whereas *N*. *spumigena* predominates in the more saline areas^9,10^.

In general, salt shock inhibits proper functioning of several proteins and metabolic processes such as photosynthesis, central metabolism, and cellular growth, clearly requiring the reconstruction of cellular metabolism^32–40^. For example, nitrogen fixation is an energy-demanding process for diazotrophic cyanobacteria^38^, and thus reducing expression of the nitrogen fixation (*nif*) gene cluster decreased the energy requirement of *Dolichospermum* sp. UHCC 0315 under stressful conditions. The transcript accumulation of chaperones, the molecules that play crucial roles in protein folding, protection, and repair under stressful conditions, were heavily modified. The role of chaperones in maintaining protein integrity under low and high salt stress was previously described^35,41^, and this study further showed the demand for different types of chaperones in salinity stress. In this study, the expression of group 2 sigma factors was heavily repressed in *Dolichospermum* UHCC 0315 growing at high salinity. The question of which sigma factors participate in gene expression in changing salinities remains unanswered^42^.

The genomes of cyanobacteria are highly dynamic entities. One factor affecting genome composition and especially the responses to phage attacks are CRISPR/Cas systems. Therefore, the finding of an unusual CRISPR repeat-spacer array lacking any known *cas* gene is of interest. Possible orphan repeat-spacer arrays exist in many filamentous cyanobacteria^43^. However, just next to this array is a gene encoding a 696 amino acid protein with similarity to TnpB transposases of the IS605 family, a feature associated also with CRISPR effector proteins Cas12b (C2c1) and C2c3^44^. The possibility that this array is part of a functional CRISPR system is supported further by our finding that spacer 7 likely targets a phage antirepressor gene present in at least two other Nostocales.

The induction of either general or stress-specific genes and regulatory systems enables cyanobacteria to thrive, even in changing environments. The transcriptional regulatory system of model cyanobacteria for adapting to elevated salt concentrations is induced rapidly^31^, but complete acclimation of metabolic processes resulting in inhibition of cell division requires longer periods of time^33,34^. For example, a study of *Synechocystis* sp. PCC 6803 showed that the number of differentially expressed genes peaked after 30 min of salt shock, and the majority of these genes returned to the control level after acclimation for 24 h^35^. Relying on the number of differentially expressed genes found after 16 days of incubation in different salt concentrations, we assumed that reconstruction of the transcriptional pattern remained vigorous long enough to minimize the harmfulness of unfavorable salt conditions and fine-tune the metabolic patterns and structural components. Accumulation and production of compatible solutes together with ion exchange through the cell membrane are major salt-stress adaptation strategies for minimizing the harmfulness of increased intracellular ion concentrations^36^. Of the compatible solutes, low-halotolerance strains are able to produce trehalose and sucrose^37^. However, only genes for sucrose synthesis were found in the genome of *Dolichospermum* sp. UHCC 0315 but increased abundance of the transcripts was not identified.

Cyanobacterial genomes harbor a wide variety of gene clusters responsible for the production of biologically active natural products, of which toxins, e.g. microcystins, are the most studied molecules, due to their harmfulness to mammals. A search of the gene clusters responsible for the production of natural products in *Dolichospermum* sp. UHCC 0315 resulted in similar genomic construction to that found in the genome of *Dolichospermum* sp. UHCC 0090^27^. However, while the anabaenopeptin gene cluster was absent in the genome of the later, the hassallidin was absent in the former. The concentration of microcystin/cell produced by *Dolichospermum* sp. UHCC 0315 increased at moderate salinity (6 g L^−1^ NaCl), which is well in line with previous studies in which high salinity triggered microcystin production in *Dolichospermum*^19^. Moreover, salt shock may cause oxidative stress in cyanobacteria which is harmful especially to lipids, and activation of lipid metabolism in the target strain cells was also found^45,46^. Despite the role of toxins in cyanobacteria being partially unclear, their function as protective elements against oxidative stress has been proposed^47,48^. Our findings thus further indicated that unfavorably high salinity induces oxidative stress and, together with other cellular stress responses, microcystins may play a role as protective molecules against oxidative stress.

## Conclusion

The present study provides new insight into the genetic diversity of the *Dolichospermum* genus and explores the phylogenetic relationship of this toxic bloom-forming cyanobacterium with other members of the *Anabaena* and *Aphanizomenon flos-aquae* taxa. Despite the distinct morphological aspects of these cyanobacteria, the results support previous studies suggesting a demand for an explicit revision of these taxa. Beyond that, the study of *Dolichospermum* sp. UHCC 0315 genome revealed adaptive responses employed by the strain to explore different salinities. Future alteration in the Baltic Sea water due to climate change will possibly lead to an expansion of blooms dominated by *Dolichospermum* over *Nodularia spumigena*.

## Methods

### Strains, cultivation, and toxin analysis

Microcystin-producing *Dolichospermum* sp. strain UHCC 0315 (former name *Anabaena* sp. strain 315) is maintained at the University of Helsinki, Faculty of Agriculture and Forestry, Department of Microbiology Culture Collection (HAMBI, UHCC) under continuous illumination of 3.2–3.7-μmol photons m^−2^ s^−1^. The strain was isolated in August 1997 from a cyanobacterial bloom in the coastal Helsinki area^9^, made axenic, and since isolation cultivated in Z8 medium without nitrogen^49^. Three replicates were cultivated in four different saline solutions (0, 3, 6, and 9 g L^−1^ NaCl) at 20 °C under continuous illumination of 3.2–3.7-μmol photons m^−2^ s^−1^ for 24 d. Chlorophyll *a* was measured at 4-d intervals by filtering 1 mL of culture through 21-mm GF/C glass microfiber filters (GE HealthCare, Chicago, IL, USA). Chlorophyll *a* was extracted using 1 mL of 90% acetone, and absorbance was determined at 664, 647, and 630 nm and calculated as described earlier^50^. Cell counting was applied accordingly to Coloma *et al*.^51^. The combined intra- and extracellular concentrations of microcystins were determined using LC-MS as described earlier^23^. A standard curve containing a dilution series of known microcystin concentrations was run alongside the toxin samples.

### Genome sequencing and annotation

A NucleoBond^®^ anion-exchange (AXG) kit (Macherey-Nagel GmbH & Co. KG, Düren, Germany) was used to extract high-molecular-weight genomic DNA from the middle-logarithmic growth phase of *Dolichospermum* sp. UHCC 0315. The DNA libraries, PacBio RS II sequencing with P6-C4 chemistry, and genome assembly using Hierarchical Genome Assembly Process 3 (HGAP3) protocol with Quiver polishing^52^ were conducted in the DNA Sequencing and Genomics Laboratory, Institute of Biotechnology, University of Helsinki. Paired-end Illumina Hiseq2500 reads (Macrogen Inc., Seoul, South Korea) were mapped twice against the PacBio assembly, using Pilon v. 1.20 software and resulting in correction of 225 sites^53^.

The genome of *Dolichospermum* sp. UHCC 0315 was annotated, using Prokka v. 1.12^54^ and noncoding RNA (ncRNA) by querying the RNA family (rfam) database^55^, using cmscan from Infernal^56^. An additional automatic annotation and functional classification in subsystems were performed using the default parameters in the RAST server^57^ and SEED viewer^58^. InterProScan 5 was used to polish the protein annotation, followed by manual curation in the genome browser Artemis^59^. Blast KEGG Orthology And Links Annotation (BlastKOALA) v. 2.1^60^ was used to annotate the KEGG Orthology (KO) orthologs and Kyoto Encyclopedia of Genes and Genomes (KEGG) pathways. Clusters of Orthologous Groups (COG) orthology was annotated by querying the National Center for Biotechnology Information (NCBI) Conserved Domain Database (CDD)^61^ with an e-value cutoff of 1e-5, using Reversed Position Specific BLAST (RPSBLAST) 2.2.31+^62^. BLAST2GO^63^ was used to annotate the Gene Ontology (GO) term. The putative secondary metabolites and biosynthetic gene clusters were predicted, using the antiSMASH 3.0 online server^64^. The genome and plasmid sequences were scanned against the Clustered Regularly Interspaced Short Palindromic Repeat database (CRISPRdb), using CRISPRfinder and CRISPRCasFinder to annotate the CRISPR loci^65–67^. The Insertion Sequence (IS) elements were annotated, using the IS Semiautomatic Genomic Annotation (ISsaga) webserver^68^.

To ensure two genetic substrains, a total of 70 single filaments of *Dolichospermum* UHCC 0315 were extracted on Z8X agar plates and further cultivated in liquid Z8X medium^49^. DNA was extracted from three surface-growing and three bottom-growing cultures, using the Macherey-Nagel NucleoSpin kit. The PCR primers were designed to amplify the 300-bp region of the deleted gene BMF77_01254, as well as three regions in plasmids 1–3 (Supplementary Table S1). The PCR mixture contained 1 × DyNAzyme PCR buffer, 0.2 mM deoxynucleoside triphosphates (dNTPs), 0.5 µM primers, and 0.5 U DyNAzyme II polymerase in a total volume of 20 µL. As a template, 50–70 ng of genomic DNA was used. DNA from the original *Dolichospermum* sp. UHCC 0315 culture was used as a positive control and water as a negative control. The PCR program was 94 °C for 3 min; 30 cycles of 94 °C for 1 min, 56 °C for 1 min, and 72 °C for 1 min; and 72 °C for 10 min. PCR amplification products were visualized on 1% agarose gel electrophoresis.

### Phylogenetic inference and comparative genomics

A maximum-likelihood phylogenomic tree was constructed by the concatenation of 31 universal marker genes^69,70^. In brief, the profiles of these marker genes were scanned against the open reading frames (ORFs) of the 76 reference genome sequences and aligned using anvi’o v5.1^71^ workflow for phylogenomics. The protein substitution model LG + I + G was assigned as the best one for the 31 proteins by performing BIC calculation in ProtTest v3.2^72^ (default parameters). A maximum-likelihood tree was estimated with Randomized Axelerated Maximum Likelihood (RAxML) v. 8.2.10^73^, based on selected partitions and substitution models, with 1000 rapid bootstrap searches. *Gloeobacter violaceus* PCC 7421 was selected as an outgroup to root the phylogenomic tree. Average Amino Acid and Nucleotide Identity heatmaps were estimate using the average identity matrices protocol in the program GET_HOMOLOGUES^74,75^ and generated using a seaborn v0.9 library heatmap script^76^.

The 16S rRNA gene tree was constructed by RAxML v8.0.0^73^ with 1000 bootstrap replicate using 75 cyanobacterial strains. The genes were aligned using MEGA v10.0.5 (default parameters)^77^ and the evolutionary model GTR + I + G selected as best fitting by jModelTest v2.1.1^78^.

Pan and core-genomes were estimated using the OrthoMCL algorithm and Tettelin function implemented in the program GET_HOMOLOGUES^74,75^. OrthoVenn with default parameters was employed to generate the Venn diagram of *Dolichospermum* sp. UHCC 0315 and UHCC 0090^79^. Shared and specific proteins were extracted using the server tools and automatically annotated by the default parameters in the metagenomics analysis server (MG-RAST)^80^. Pairwise genome alignments and dotplots were performed, using MUMmer v. 3.1.^81^. Synteny blocks were predicted and visualized, using the jcvi v. 0.7.1 Python library^82^ and local synteny blocks were visualized, using the genoPlotR v. 0.8.4 package^83^.

### RNA sequencing

On day 16, cells from the cultures containing 0 and 6 g L^−1^ NaCl were collected on 0.22 µm polycarbonate filters (GE Water and Process Technologies). The filters were frozen in liquid nitrogen and stored at −80 °C. Prior to harvesting, the liquid cultures were fixed by 5% phenol in 10% ethanol. RNA was extracted, using the RNeasy mini kit (Qiagen N.V., Venlo, The Netherlands). For the depletion of genomic DNA, the TURBO DNA-*free*™ kit (Life Technologies, now ThermoFisher Scientific, Carlsbad, CA, USA) was used, and the RNA was further cleaned and concentrated with the RNA Clean & Concentrator™ kit (Zymo Research Corp., Irvine, CA, USA). RNA integrity and purity were determined, using the Bioanalyzer RNA 6000 Nano Kit (Agilent Technologies Inc., Santa Clara, CA, USA). Ribosomal RNA (rRNA) removal (MICROBExpress™ Bacterial messenger RNA (mRNA) enrichment kit, Life Technologies), complementary DNA (cDNA) library preparation (Bacterial ScrptSeq Complete Kit, Illumina), and paired-end Illumina Hiseq2500 sequencing were carried out at the Institute for Molecular Medicine Finland (FIMM).

The quality of the raw reads was checked using FastQC v. 0.11.4 (Babrahan Bioinformatics, Babrahan Institute, Cambridge, UK)^84^, and the reads were trimmed with Trimmomatic^85^. The clean reads were aligned to the reference genomes, using Burrows-Wheeler Aligner-Maximum Exact Match (BWA-MEM) v. 0.7.7^86^ in paired-end mode with default parameters. Sequence Alignment/Map tools (SAMtools) v. 1.2^87^ was applied to convert the resulting SAM format to the Binary Alignment Map (BAM) format and filtered with the BAM filter (Galaxy Version 0.5.7.1) to remove the reads that were unmapped, less than 20 nucleotides long, flagged as secondary alignments, marked as PCR duplicates, or of low quality. The filtered BAM files were sorted with chromosomal coordinates using SAMtools v. 1.19^87^ and converted to Wiggle format, using the script bam2wig.py from the RNA Sequence Quality Control (RseQC) v. 2.4 package^88^ in paired-end mode, and normalized to 1000000000 wigsum. The Wiggle format was then converted to Artemis^59^-compatible genome coverage graphs, using custom scripts available at https://github.com/housw/GRPutils. FeatureCounts v. 1.4.6.p5^89^ was used to quantify unambiguously aligned fragments. Only paired-end reads that constituted a fragment size within 50~600 nucleotides long were considered, and chimeric fragments were excluded. The Galaxy instance of Freiburg University was used for RNA-Seq analysis^90,91^.

To exclude weakly expressed features, the counts were converted to counts per million (CPM); only features having a higher CPM (sum of CPM across all samples should be more than 3 and at least three samples had a CPM more than 1) were taken for differential expression analysis. Features belonging to rRNA and transfer RNA (tRNA) were excluded in this study. The differentially expressed genes between control and treatment were called, using both edgeR v3.14.0^92^ and DESeq v1.9.12^25^ following the simple design protocol^93^. A GO enrichment test was performed for the differentially expressed genes, using GOstats v. 2.40.0^94^ with all the expressed genes as background. Semantic similarities of the enriched GO terms were calculated, using the REVIGO online server^95^.

## Supplementary information

Supplementary Material

Table S2

## Data Availability

The sequences were deposited in the NCBI database under the BioProject accession number PRJNA377208.
