# Supplementary material for: Insight into the genome and brackish water adaptation strategies of toxic and bloom-forming Baltic Sea Dolichospermum sp. UHCC 0315
Source: Sci Rep. 2019 Mar 20;9:4888. doi: 10.1038/s41598-019-40883-1 (PMC6426976; doi:10.1038/s41598-019-40883-1)
Supplement: Supplementary file 1 — Supplementary Material [file 41598_2019_40883_MOESM1_ESM.pdf]

Insight into the genome and brackish water adaptation strategies of toxic and bloom-forming Baltic Sea *Dolichospermum* sp. UHCC 0315

Authors: Jonna E. Teikari[1], Rafael V. Popin[1], Shengwei Hou[2], Matti Wahlsten[1], Wolfgang R. Hess[2] and Kaarina Sivonen[1*]

[1] Department of Microbiology, University of Helsinki, Viikinkaari 9, FI-00014 University of Helsinki, Finland
[2] Genetics & Experimental Bioinformatics, Institute of Biology III, University Freiburg, Schänzlestraße 1, D-79104 Freiburg, Germany

[*]Corresponding author
E-mail: kaarina.sivonen@helsinki.fi

Supporting information

Figure S1. Putative CRISPR repeat-spacer array in *Dolichospermum* sp. UHCC 0315. Direct repeat sequences are highlighted in pink (a). Spacer 7 (highlighted in green) exhibits extensive sequence identity to prophage antirepressor sequences in Nostocales genomes (b,c).

a) Dolichospermum sp. UHCC 0090 hypothetical CRISPR sequence

```
CTTGCAATTAACCTAATTACTCAAAGCTAATTTCACC   CAGTAAAGGATGCAGTTAGGGAACAATCTTCTGGAC
CTTGCAATTAACCTAATTACTCAAAGCTAATTTCACC   TTCATCTACAGTACAGCCGCAACTATTCCGCGTACTC
CTTGCAATTAACCTAATTACTCAAAGCTAATTTCACC   TTTTCTATCTAGACTACGACATCAACCTAAAAC
CTTGCAATTAACCTAATTACTCAAAGCTAATTTCACC   TAATCTTTTGGTGAGTTTCCTGTAGGGTTCTAGG
CTTGCAATTAACCTAATTACTCAAAGCTAATTTCACC   CCAGCACCTTCCCTTTTACCACCTTTATTCATAA
CTTGCAATTAACCTAATTACTCAAAGCTAATTTCACC   TTATTCCCCCCGTTTTTCTGGAGGGGTTAACA
CTTGCAATTAACCTAATTACTCAAAGCTAATTTCACC   ATTTTCTGTAGTCCACCAGGGGTCATAATTTGTTTAT
CTTGCAATTAACCTAATTACTCAAAGCTAATTTCACC   CCCTTCCCAGCGGTGGCATAGGGTATGGGATAA
CTTGCAATTAACCTAATTACTCAAAGCTAATTTCACC   TTTCAGCTTTAGCATCGGCTAATGTGCCAGAA
CTTGCAATTAACCTAATTACTCAAAGCTAATTTCACC   TTGGCTGGCAATTCCAATTCGATGGGAATTGAACC
CTTGCAATTAACCTAATTACTCAAAGCTAATTTCACC   TCATGGCTGCACTCGGTTCTCAATTGAGTCAGAT
CTTGCAATTAACCTAATTACTCAAAGCTAATTTCACC   AACTCGCAGAGGGGGGATGCTAGTGGACTACAAA
CTTGCAATTAACCTAATTACTCAAAGCTAATTTCACC
```

b) Alignment of the spacer 7 to a prophage antirepressor sequence in *Nostoc* sp. 'Peltigera membranacea cyanobiont' N6:

```
Spacer 7       1  ATTTTCTGTAGTCCACCAGGGGTCATAATTTGTTTAT  37
                  ||||||||   ||| ||||||||||||||| ||||||
Sbjct    2328806  ATTTTCTGCTTTCCGCCAGGGGTCATAATTGGTTTAT  2328842
```

c) Alignment of the spacer 7 to a prophage antirepressor sequence in *Nostoc flagelliforme* CCNUN1:

```
Spacer 7       1  ATTTTCTGTAGTCCACCAGGGGTCATAATTTGTTTAT  37
                  ||||||||   ||| ||||||||||||||| ||||||
Sbjct    7643473  ATTTTCTGCTTTCCGCCAGGGGTCATAATTGGTTTAT  7643437
```

Figure S2. Full-length gel with PCR amplification of the region which is absent in the filament growing at the bottom of the flasks shown in Figure 1e.

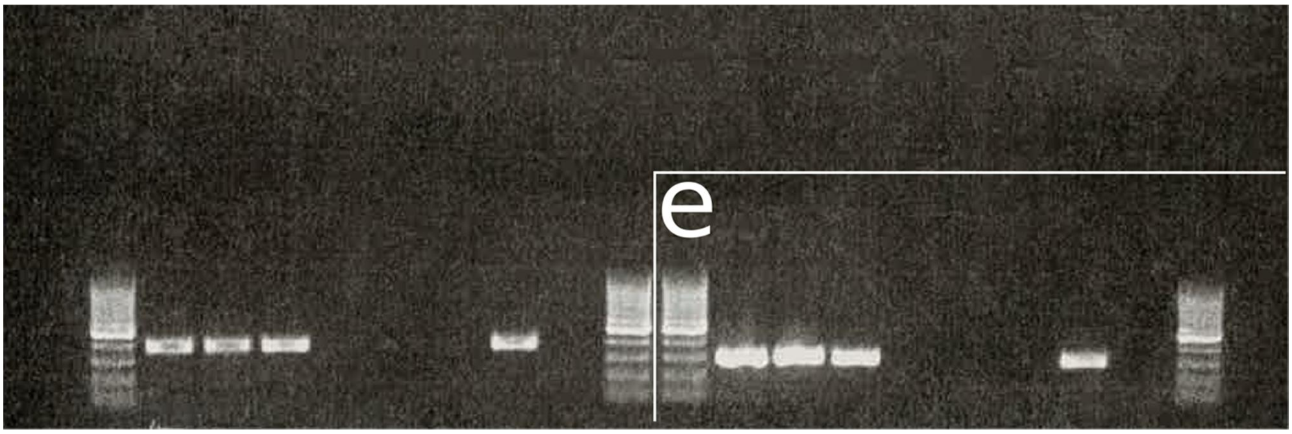

Figure S3. Full-length gel with PCR amplification of the three plasmids shown in Figure 1f-h.

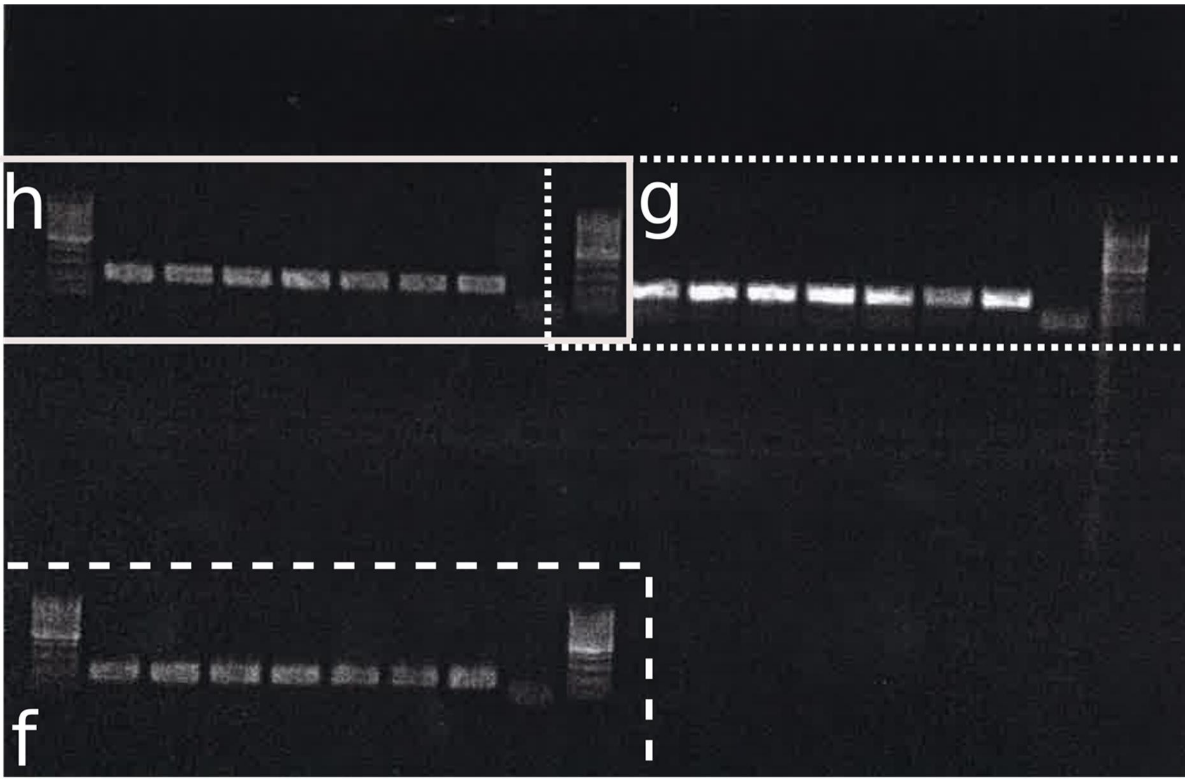

Figure S4. 16S rRNA gene Bayesian inference tree constructed with a total of 76 cyanobacterial strains. Genomes analyzed in the present study are colored according to subgroups (Iα- δ, II and III) and those included in the *Anabaena/Dolichospermum/Aphanizomenon* clade are in bold. Accession numbers are presented within parentheses.

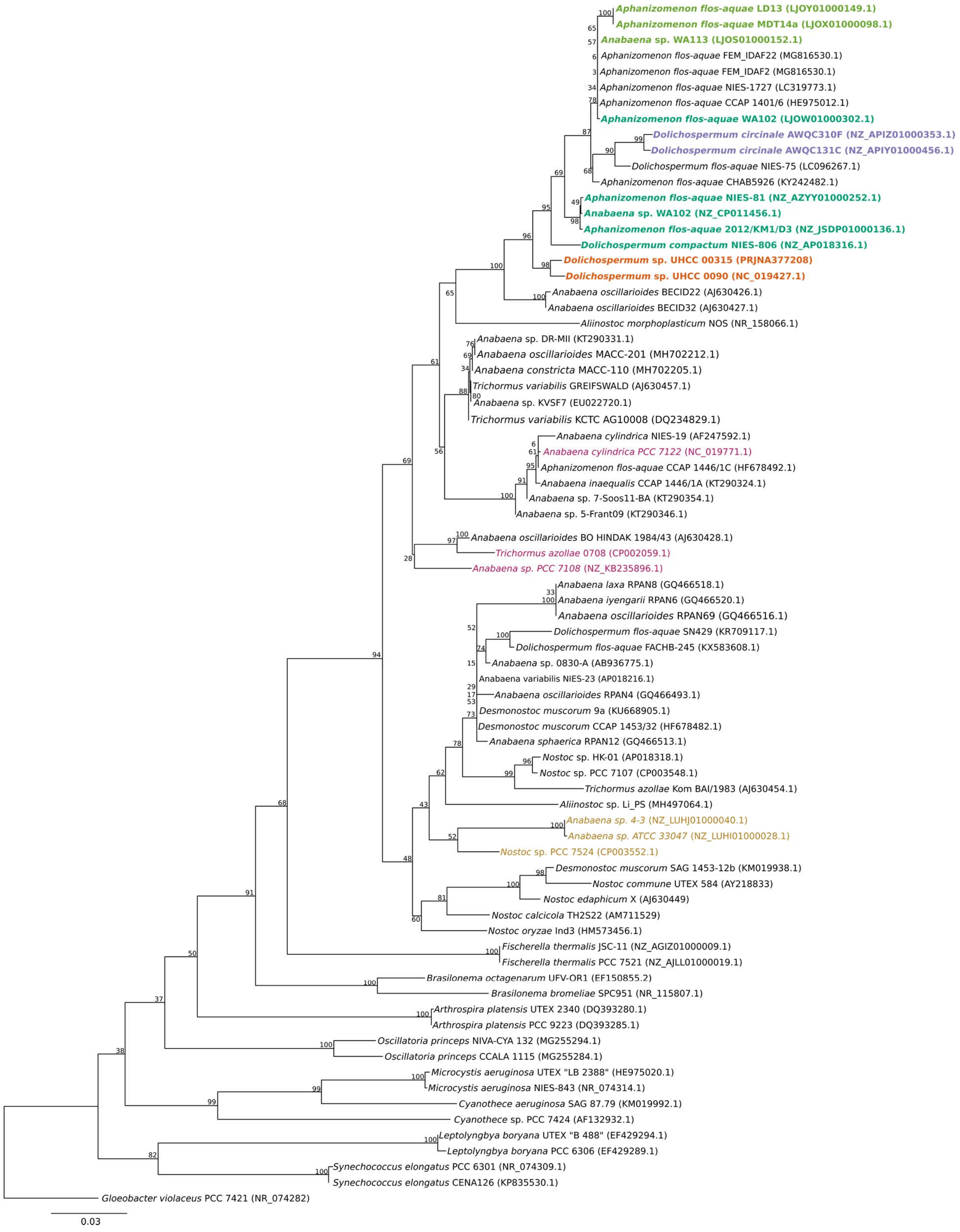

Figure S5. Synteny of sequenced *Dolichospermum* sp. UHCC 0315 and closest counterpart UHCC 0090.

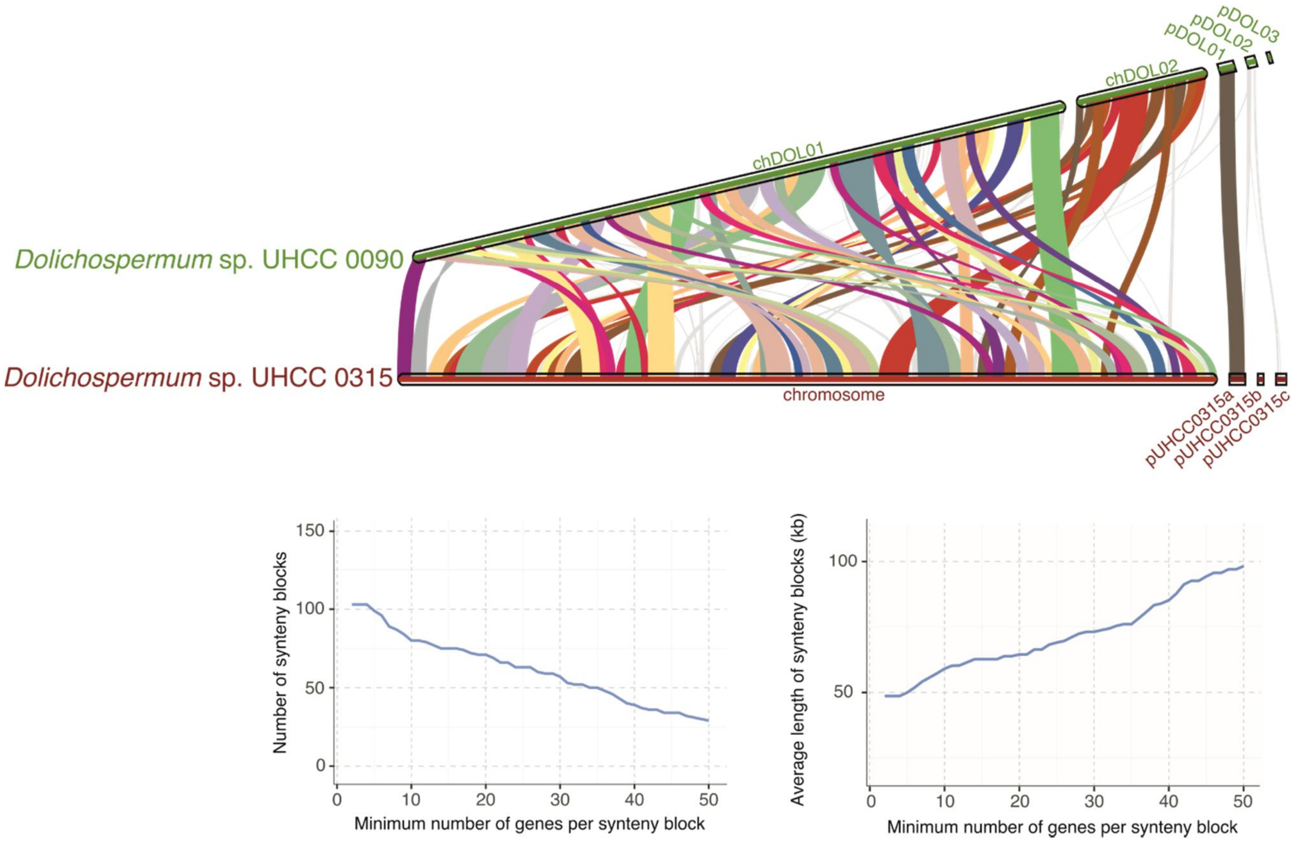

Figure S6. Venn diagram of *Dolichospermum* sp. UHCC 0315 and UHCC 0090. Both the specific and shared proteins were automatically annotated and classified in subsystems.

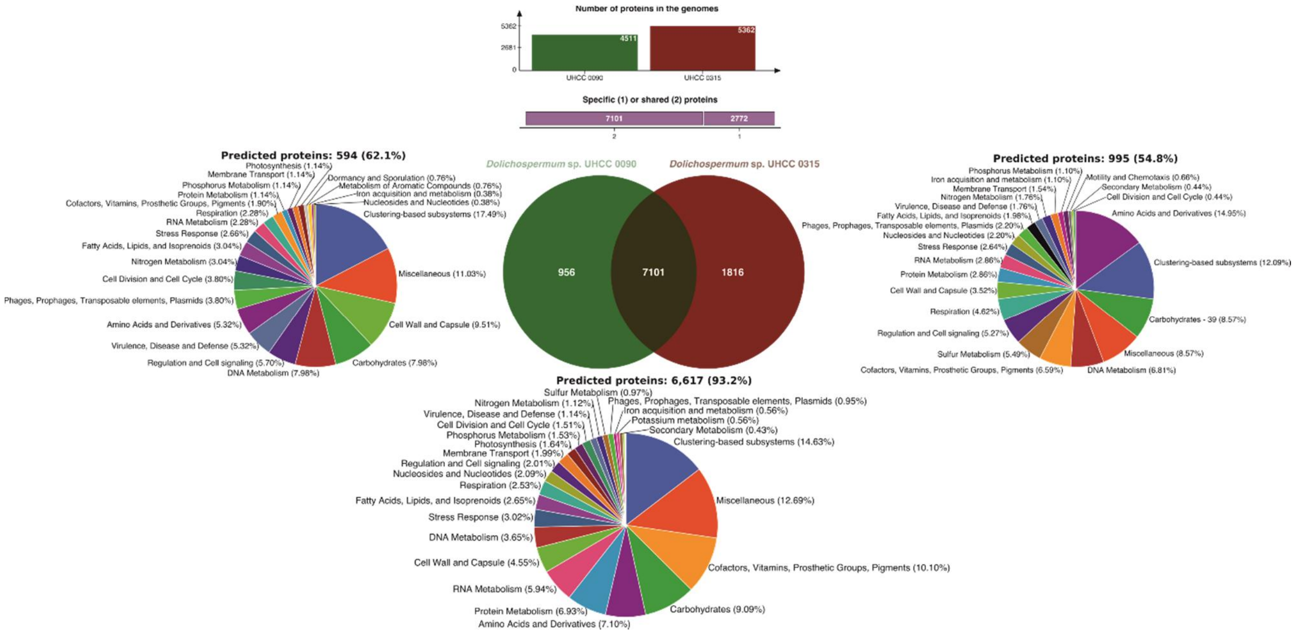

Figure S7. Comparison of the Gene Ontology (GO) enrichment analysis of the up-regulated (a) and down-regulated (b) genes of *Dolichospermum* sp. UHCC 0315 in two different salinity conditions (0 g L^{-1} and 6 g L^{-1} of added NaCl). Circle sizes represent the number of expressed genes (FDR≤ 0.01) while the color represents the $\log_{10}$ adjusted *p*-value under hypogeometric test. GO terms with dispensability smaller than 0.15 are highlighted.

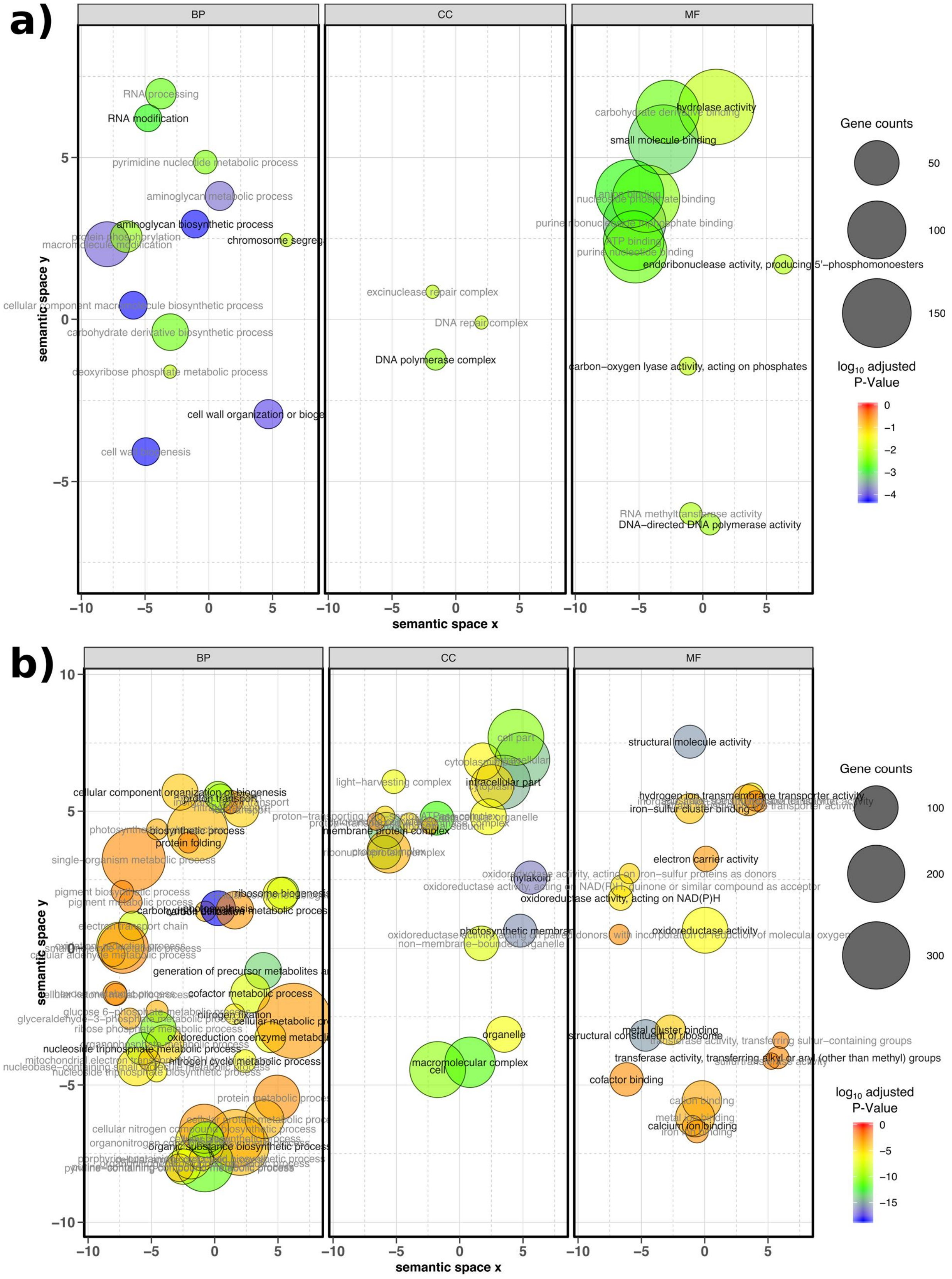

Figure S8. Microcystin production by *Dolichospermum* sp. UHCC 0315 in two different salinities (0 and 6 gL^{-1} of NaCl). a) Quota of microcystins (fg mcy $cell^{-1}$) and b) concentration of the total microcystins in experiment (ng/mL). Standard error bars are represented in both a) and b).

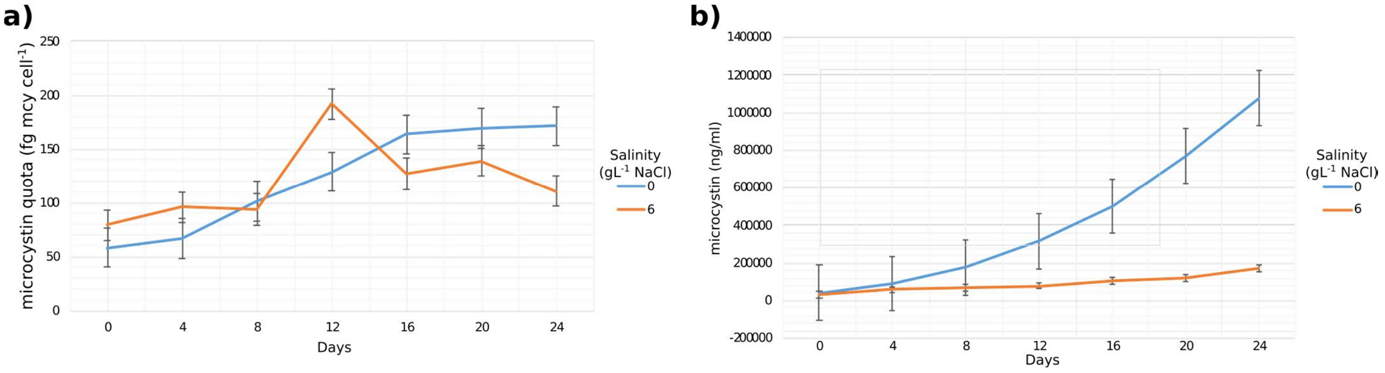

Table S1. Sequences, product size and melting temperatures of the primers used in the PCR amplification studies of the BMF77_1250-1254 region and the plasmids.

Target gene	Location	Primer	Seuqence 5'->3'	Pruduct size (bp)	Melting temperature (°C)
BMF77_1254	Deletion site	BMF77_1254F	ACAGACTTCAACAGGCCACA	300	58
		BMF77_1254R	AAAGGGCAGTAAGGGTGGAT		58
BMF77_04804	Plasmid 1	BMF77_04804F	TTGGTAGATGCTCCAGTGGG	225	60
		BMF77_04804R	TAGAGCGTTTTCCAGTCCCT		58
BMF77_04902	Plasmid 2	BMF77_04902F	CCCATTGCCAACCTGTTTCA	200	58
		BMF77_04902R	GCGGTGAAAGATGAGGTGTC		60
BMF77_04929	Plasmid 3	BMF77_04929F	TCAAAGACCCCACAGAAGCT	167	58
		BMF77_04929R	ATATGGCTTGAGTCCACCGC		60

Table S2. Gene expression of *Dolichospermum* sp. UHCC 0315 triplicates at 0 and 6 gL^{-1} of added NaCl.

Table S3. Gene clusters annotation of the bioactive compounds identified in the genome of *Dolichospermum* sp. UHCC 0315.

	Gene ID	Gene name	Product
Microcystin	BMF77_03385	*mcyC*	non-ribosomal peptide synthetase
	BMF77_03386	*mcyB*	non-ribosomal peptide synthetase
	BMF77_03387	*mcyA*	McyA protein
	BMF77_03388	*mcyG*	peptide synthetase polyketide synthase fusion protein McyG
	BMF77_03389	*mcyD*	polyketide synthase
	BMF77_03390	*mcyJ*	methyltransferase
	BMF77_03391	*mcyE*	hybrid non-ribosomal peptide synthase/polyketide synthase
	BMF77_03392	*mcyF*	Asp/Glu racemase McyF
	BMF77_03393	*mcyI*	dehydrogenase McyI
	BMF77_03394	*mcyH*	ABC transporter ATP-binding protein
Anabaenopeptilide	BMF77_03804	*adpG*	ABC transporter ATP-binding protein
	BMF77_03805	*adpF*	short-chain dehydrogenase/reductase ApdF
	BMF77_03806	*adpE*	methyltransferase ApdE
	BMF77_03807	*adpD*	anabaenopeptilide synthetase ApdD
	BMF77_03808	*adpC*	halogenase ApdC
	BMF77_03809	*adpB*	anabaenopeptilide synthetase ApdB
	BMF77_03810	*adpA*	anabaenopeptilide synthetase ApdA
Anabaenopeptin	BMF77_02332	*aptF*	ABC transporter ATP-binding protein
	BMF77_02333	*aptE*	aptE protein
	BMF77_02334	*aptD*	non-ribosomal peptide synthetase
	BMF77_02335	*aptC*	non-ribosomal peptide synthetase
	BMF77_02336	*AptB*	non-ribosomal peptide synthetase
	BMF77_02337	*aptA2*	non-ribosomal peptide synthetase
	BMF77_02338	*aptA1*	non-ribosomal peptide synthetase

Anacyclamide	BMF77_00862	*acyC*	Anacyclamide biosynthesis protein
	BMF77_00863	*acyB*	Anacyclamide biosynthesis protein
	BMF77_00864	*acyA*	subtilisin-like protease
	BMF77_00865		Anacyclamide precursor
	BMF77_00866		Anacyclamide precursor
	BMF77_00867		Transposase
	BMF77_00868	*acyF*	Anacyclamide synthesis protein
	BMF77_00869		Hypothetical protein
	BMF77_00870		Hypothetical protein
	BMF77_00871		fatty-acid oxidation protein subunit alpha
	BMF77_00872		fatty-acid oxidation protein subunit alpha
	BMF77_00873	*acyG*	subtilisin-like protease
Putative group I bacteriocin gene cluster	BMF77_01231		Peptidase C39
	BMF77_01232	*hlyD*	Secretion protein HlyD
	BMF77_01233		peptidylprolyl isomerase
	BMF77_01234		hypothetical protein
	BMF77_01235		aminoglycoside phosphotransferase
	BMF77_01236		Hypothetical protein
	BMF77_01237		Cyanophysin synthase
	BMF77_01238		Hypothetical protein
	BMF77_01239		Peptidase M3
Putative group I bacteriocin gene cluster	BMF77_04209		peptidyl-prolyl cis-trans isomerase
	BMF77_04210		Peptidase C39
	BMF77_04211	*hlyD*	secretion protein HlyD
Putative group III bacteriocin gene cluster	BMF77_00924		hypothetical protein
	BMF77_00925		hypothetical protein
	BMF77_00926		Peptidase C39
	BMF77_00927	*HlyD*	HlyD family secretion protein
Putative unclassified group bacteriosin gene cluster	BMF77_00718		peptidylprolyl isomerase
	BMF77_00719		Peptidase C39
Putative group I bacteriocin gene cluster	BMF77_00562		hypothetical protein
	BMF77_00563		Transposase
	BMF77_00564		Peptidase C39
	BMF77_00565	*hlyD*	secretion protein HlyD
	BMF77_00566		Hypothetical protein
	BMF77_00567		Hypothetical protein
Putative group III bacteriocin gene cluster	BMF77_01905		Hypothetical protein
	BMF77_01906		Hypothetical protein
	BMF77_01907		Hypothetical protein
	BMF77_01908		Hypothetical protein
	BMF77_01909		Hypothetical protein
	BMF77_01910		ABC transporter ATP-binding protein
	BMF77_01911	*hlyD*	secretion protein HlyD
